# A Decade of Boon or Burden: What Has the CHIP Ever Done for Cellular Protein Quality Control Mechanism Implicated in Neurodegeneration and Aging?

**DOI:** 10.3389/fnmol.2016.00093

**Published:** 2016-10-04

**Authors:** Vibhuti Joshi, Ayeman Amanullah, Arun Upadhyay, Ribhav Mishra, Amit Kumar, Amit Mishra

**Affiliations:** ^1^Cellular and Molecular Neurobiology Unit, Indian Institute of Technology JodhpurRajasthan, India; ^2^Centre for Biosciences and Biomedical Engineering, Indian Institute of Technology IndoreMadhya Pradesh, India

**Keywords:** E3 ubiquitin ligases, misfolded proteins, neurodegeneration, cell death, aging

## Abstract

Cells regularly synthesize new proteins to replace old and abnormal proteins for normal cellular functions. Two significant protein quality control pathways inside the cellular *milieu* are ubiquitin proteasome system (UPS) and autophagy. Autophagy is known for bulk clearance of cytoplasmic aggregated proteins, whereas the specificity of protein degradation by UPS comes from E3 ubiquitin ligases. Few E3 ubiquitin ligases, like C-terminus of Hsc70-interacting protein (CHIP) not only take part in protein quality control pathways, but also plays a key regulatory role in other cellular processes like signaling, development, DNA damage repair, immunity and aging. CHIP targets misfolded proteins for their degradation through proteasome, as well as autophagy; simultaneously, with the help of chaperones, it also regulates folding attempts for misfolded proteins. The broad range of CHIP substrates and their associations with multiple pathologies make it a key molecule to work upon and focus for future therapeutic interventions. E3 ubiquitin ligase CHIP interacts and degrades many protein inclusions formed in neurodegenerative diseases. The presence of CHIP at various nodes of cellular protein-protein interaction network presents this molecule as a potential candidate for further research. In this review, we have explored a wide range of functionality of CHIP inside cells by a detailed presentation of its co-chaperone, E3 and E4 enzyme like functions, with central focus on its protein quality control roles in neurodegenerative diseases. We have also raised many unexplored but expected fundamental questions regarding CHIP functions, which generate hopes for its future applications in research, as well as drug discovery.

## Introduction

A nascent polypeptide chain achieves its native structure with the help of folding machinery present inside the cells that includes molecular chaperones and other associated proteins. However, many of these proteins are not able to fold properly and are needed to be cleared from the cell by the cellular protein quality control system that includes mainly ubiquitin proteasome system (UPS) and autophagy. These two systems either work independently or in collaboration with each other (Goldberg, 2003; Korolchuk et al., 2010). Molecular chaperones are the supervisors of newly synthesized polypeptides in the crowded cellular *milieu*. The guidance of chaperones assists immature polypeptides to acquire a functional macromolecular structure and move towards appropriate cellular localization to perform pre-defined functions (Ellis and Hemmingsen, 1989; Hartl, 1996). Two well-studied chaperones are heat shock proteins, Hsp70 and Hsp90. Heat shock proteins function as stress-induced proteins to monitor changes in the molecular environment of the cell (Nollen and Morimoto, 2002). The chaperones not only assist new proteins in achieving the functional activity by proper folding, but also play crucial roles in removal of aberrant proteins, either by UPS or by autophagy pathway (Hartl et al., 2011).

**Figure 1:**
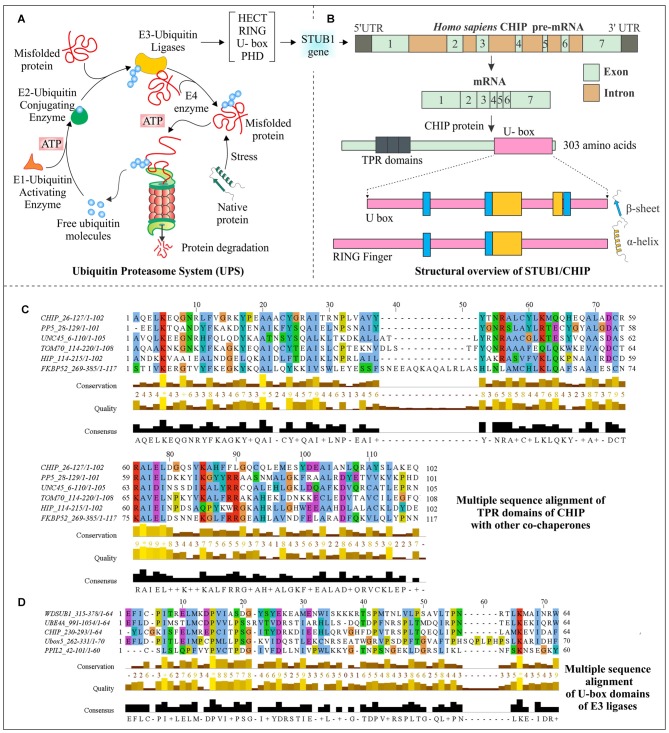
**Structural aspects of C-terminus of Hsc70-interacting protein (CHIP) and multiple sequence analysis of its domains. (A)** Schematic representation of the ubiquitin proteasome system (UPS) shows an ATP-dependent ubiquitin activation by E1 ubiquitin activating the enzyme, followed by attachment of ubiquitin to E2 conjugating enzymes and later targeting misfolded proteins by specific E3 ubiquitin ligase enzymes for degradation by 26S proteasome complex. **(B)** Human STIP1 Homology And U-Box Containing Gene 1 (STUB1) gene codes for E3 ubiquitin ligase CHIP; mRNA contains seven exons and six introns. The mRNA synthesizes a 303 amino acid long protein, which contains three tetratricopeptide repeat (TPR) domains at its N-terminal and one U-box domain at its C-terminals. A structural comparison of U-box domain with RING finger domain has been done to illustrate the respective arrangements of α-helices and β-sheets. **(C)** Sequence alignment of CHIP with other co-chaperones, showing evolutionarily conserved TPR domains. **(D)** U-box domains of CHIP and different E3 ubiquitin ligases have been aligned to elucidate their conserved region. Clustal Omega and Jalview were used for alignment and visualization respectively, with default coloring schemes (Clamp et al., 2004; Sievers et al., 2011). Asterisk (*) shows absolute conservation of amino acids, whereas score 0–9 followed by plus sign shows the levels of amino acid conservation.

Chaperones participate in protein quality control processes of the cell by forming complexes with E3 ubiquitin ligases, ubiquitin molecules, various accessory co-chaperones (Hsp40, BAG-1, BAG-3, etc.) and other proteins to tag misfolded or improperly folded proteins for their removal from the cells (Edkins, 2015). UPS involves ubiquitin-dependent degradation of various misfolded proteins (Ciechanover, 1994; Hochstrasser, 1996). UPS consists of three enzymes E1 ubiquitin activating enzyme, E2 ubiquitin conjugating enzymes and E3 ubiquitin ligases. Other than these enzymes a small 76 amino acid long ubiquitin protein, and 26S proteasome, the proteolytic machinery of the cell with different types of protease activities, are other major components of the UPS (Hershko, 1998). As shown in Figure 1A, ubiquitination of a particular protein, with the help of successive activities of E1, E2 and E3 enzymes generates a degradation signal onto a protein, for its translocation to the 26S proteasome interior, where the catalytic sites of proteasome cleave polypeptide into smaller oligopeptides. The specificity in the functioning of UPS comes from E3 ubiquitin ligase enzymes, which recognize misfolded proteins and continue their elimination through catalytic activities of 26S proteasome (Pickart, 2001).

Autophagy is another widely explored pathway that helps in clearance of the cytoplasmic bulk of misfolded proteins, by using lysosomal proteolysis (Seglen and Bohley, 1992). The cytoplasmic bulk includes aggregates, oligomers, protein complexes, misfolded soluble proteins and sometimes cellular organelles. Autophagic degradation begins with the formation of the double-layered membrane, the phagophore, that is designated as autophagosome when wrapped around autophagic substrates. The tag of autophagosomal markers, like p62 helps in the transportation of autophagosome to lysosome for final degradation by lysosomal hydrolases (Korolchuk et al., 2010). Chaperone-assisted selective autophagy (CASA) and chaperone-mediated autophagy (CMA) are examples of two types of autophagy, where chaperones are involved in protein degradation, using this lysosomal pathway (Kaushik and Cuervo, 2012). Chaperone-assisted proteasomal degradation (CAP) elucidates another mechanism where chaperones steer misfolded proteins towards the ubiquitin-mediated proteasomal degradation (Kettern et al., 2010). Chaperones are also responsible for the crosstalk between these two pathways of protein degradation (Park and Cuervo, 2013).

C-terminus of Hsc70-interacting protein (CHIP) is profoundly involved in most of the above-described quality control pathways (Kettern et al., 2010). Previously, we have shown various cellular strategies embraced by E3 ubiquitin ligases in the removal of aberrant cellular proteins. Few E3 ubiquitin ligases like CHIP, cullin5, E6-AP, etc. function in association with various chaperones like Hsp70, Hsp90, etc. to maintain the quality of cellular proteins (Chhangani et al., 2013). CHIP is one of the most extensively studied E3 ubiquitin ligase associated with chaperones (Murata et al., 2001). E3 ubiquitin ligase activity of CHIP is because of its evolutionarily conserved U-box domain, which is a modified form of the Really Interesting New Gene (RING) domain which also contains a metal chelating region (Hatakeyama et al., 2001; Jiang et al., 2001). Cytoplasmic 35 kDa protein CHIP was firstly recognized as a chaperone interacting protein because of its tetratricopeptide repeat containing (TPR) domain, which is similar to Hsp70-Hsp90 organizing protein (Hop) and Hsc70 interacting protein (Hip; Ballinger et al., 1999). Negative regulation of a substrate binding cycle of Hsc70-Hsp70 by CHIP reveals its dual role as co-chaperone as well as E3 ubiquitin ligase for protein quality control in cells (Connell et al., 2001). CHIP has also been shown as a connecting link between chaperones and proteasome system (McDonough and Patterson, 2003).

The involvement of CHIP in various physiological functions defines its worth in the maintenance of cellular homeostasis; earlier findings have described its roles in a number of pathways and diseases (Paul and Ghosh, 2015). However, its exceptional significance was found in an array of neurodegenerative disorders with greater extent (Dickey et al., 2007). Another potential reason of extensive research on CHIP is its association with a wide range of diseases, such as cardiac disorders, muscular disorders and different types of cancers (Adachi et al., 2007; Willis et al., 2008; Cao et al., 2016). In this review, we first describe structural aspects and involvement of CHIP in various quality control pathways, and then we elaborate its liaison in various diseases, with a special focus on neurodegeneration and associated disorders. Loss of neurons is more crucial than the loss of any other cell in the body, as the possibility of recovery or replacement is negligible in this case (Currais et al., 2009). Understanding and analyzing available data about molecules like CHIP, that are directly linked to multiple neurodegenerative diseases, will provide hope for the development of preventive measures for these pathologies. We, here, try to compile a detailed and descriptive analysis of CHIP and its association with neurodegenerative disorders, to focus on its wide scope in future drug discovery.

## Journey of Chip: Gene to Protein

The CHIP protein in humans is encoded by STIP1 Homology and U-Box Containing gene 1 (STUB1), with the cytogenetic band at location p13.3 on chromosome number 16. The pre-mRNA consists of seven exons and six introns within 2760 base pairs of the CHIP/STUB1 gene. As represented in Figure 1B, human CHIP protein is of 303 amino acids in length (Ballinger et al., 1999; Shi et al., 2013). The first identification of CHIP antigen was done in chronic lymphocytic leukemia patients, using serologic identification by recombinant expression cloning (SEREX) method (Krackhardt et al., 2002). The two isoforms produced by alternate splicing are of 303 and 231 amino acid long sequences having NCBI accession: NP_005852.2 and NP_001280126.1 respectively, but no experimental confirmation for the second isoform is available.

### Structural Overview of CHIP Domains

Interaction of CHIP with heat shock proteins occurs because of its TPR domain; this interaction of CHIP with C-terminus of Hsc70 or Hsp70 does not have any interference in its binding with misfolded proteins. Apart from TPR domain, its adjacent positively charged region is also significantly responsible for this interaction (Ballinger et al., 1999; McDonough and Patterson, 2003). Located at N-terminal of CHIP, this TPR domain consists of three pairs of anti-parallel α-helices and elongated seventh helix with packed N-terminus against its third helical pair (Zhang et al., 2005). TPR domains are structurally conserved among many other co-chaperones, which also have a tendency to bind with Hsp70 and Hsp90 (McDonough and Patterson, 2003). In a multiple sequence alignment analysis, which is presented in Figure 1C, conserved nature of this domain in CHIP and other co-chaperones are observed, giving an indication of how the interaction of CHIP occurs with chaperones and how this is directly associated with many other co-chaperones.

STUB1/CHIP is a member of U-box-containing E3 ubiquitin ligases family, which is also responsible for conferring it an E4 enzyme-like activity (Jiang et al., 2001). The conserved ~70 amino acids long U-box domain is found from yeasts to humans, in various proteins (Hatakeyama et al., 2001). Previously, only two families of E3 ubiquitin ligases, Homologous to the E6-AP Carboxyl Terminus (HECT) and RING were well characterized (Passmore and Barford, 2004; Metzger et al., 2012). But in last two decades, many U-box containing proteins were identified for their function in polyubiquitination (Aravind and Koonin, 2000; Ohi et al., 2003). They were therefore established as a new family of E3 ubiquitin ligases which are also involved in protein quality control functions (Cyr et al., 2002). The U-box domain was first identified as multiubiquitin chain forming domain in a yeast ubiquitin fusion degradation 2 (UFD2) protein (Koegl et al., 1999). Later, it was found that all mammalian U-box proteins interact with chaperones, as in the case of CHIP (Hatakeyama et al., 2004). Figure 1D compares conserved regions of CHIP U-box domain with other U-box containing E3 ubiquitin ligases obtained from multiple sequence alignment.

### Post-Translational Modifications of CHIP Polypeptide

Ubiquitination is one of the ways to regulate CHIP activity. Ube2W, an E2 ubiquitin conjugating enzyme, ubiquitylates CHIP at N-terminus instead of any of its lysine residues, as observed in the mass spectrometry data. This is one type of protein modification that increases its activity of ubiquitination of substrates (Tatham et al., 2013). A similar type of modification has been observed in the case of CHIP-UbcH5A complex, which shows unconventional ubiquitination by attaching polyubiquitin chain on CHIP, lacking specificity for any of the seven lysine residues of ubiquitin (Windheim et al., 2008). Another post-translational modification in CHIP polypeptide is phosphorylation at two serine residues Ser^19^ and Ser^23^ and also the presence of one auto-ubiquitination site Lys^22^ (Graf et al., 2010). Interaction of CHIP with laforin-malin complex promotes the translocation of CHIP from the cytoplasm to the nucleus upon heat shock (Sengupta et al., 2011). These examples of different types of modifications of CHIP sheds some light on importance of this protein in cellular interactions and also describes various ways, by which cell can precisely modify its major proteins to effectively deliver their crucial functions.

### Pathogenic Mutations of CHIP Associated with Disorders

Mutation in *STUB1* gene is one of the genetic causes of the Gordon Holmes syndrome, an autosomal recessive type of hereditary cerebellar ataxia in association with hypogonadotropic hypogonadism (Ronnebaum et al., 2014; Shi et al., 2014). Patients with mutations in this gene shows similar neurological phenotypes, as shown by patients with a mutation in two other genes associated with this syndrome *RNF216* and *OTUD1* (Shi et al., 2013; de Roux et al., 2016). SCA3 mice, a disease model of Machado-Joseph disease, also showed decreased levels of CHIP. This could probably be because of an increase in affinity of ataxin-3 for CHIP, which results in such decrease in CHIP levels throughout the brain (Scaglione et al., 2011). Another study showed that mutation in STUB1 affects its E3 ubiquitin ligase activity and prevents hypoxia inducible factor-1 A (HIF1A) degradation by CMA (Ferreira et al., 2013). A critical brain disease, intracranial aneurysm (IA), also involves *STUB1* mutation that contributes to vascular remodeling, related to the development of this disorder (Su et al., 2013). Study of IL-4R signaling regulation by *STUB1* reveals that its mutation is linked with lung inflammation having hypersecretion of the mucus and elevated level of serum IgE (Wei et al., 2014). These mutations establish the eminent participation of this E3 ubiquitin ligase protein in many pivotal metabolic pathways and developmental stages.

## Functional Plasticity of Chip

Till date, more than 600 E3 ubiquitin ligases of different domains, properties and activities have been identified, and search is still on (Li W. et al., 2008). CHIP is important because of its functional relevance in overall working and regulation of the cell proteins. CHIP orchestrates regulation of cellular proteins from folding to degradation by various mechanisms, including a coordinated function with molecular chaperones to refold the unfolded protein species, as well as a well-directed degradation of those, which are beyond the refolding range, via proteasome or autophagy. So, to accomplish such a wide range of responsibilities, it should be a multifaceted molecule (Connell et al., 2001). To perform this task, CHIP has evolved various ways to ubiquitinate its substrates by interaction with different E2 conjugating enzymes that form a variety of signals, according to which it decides the fate of substrates. E2s are the family of enzymes, having highly conserved ubiquitin conjugating domain. These enzymes, in combination with E3s, play an important role in determining the fate of substrate proteins, by selecting a lysine residue, on which ubiquitin moiety must be added. NMR studies provide evidence that E2 enzymes require a well conserved S-P-A motif in loop seven, which is found in many E2 enzymes, to recognize and bind with CHIP. The specificity that determines the binding of UbcH5 and Ubc13-Uev1a, the E2 ubiquitin-conjugating enzymes, with CHIP E3 ubiquitin ligase is their S-P-A motif. It also interacts with Ube2e2, the class III E2 enzyme because of this conserved S-P-A motif, providing the significance of this motif in such kind of interactions between U-box containing E3 ligases and associated E2 enzymes (Xu et al., 2008).

The mechanism of polyubiquitination by binding of Ubc13-Uev1a and UbcH5 with CHIP differ from each other as Ubc13-Uev1a association causes Lys^63^-linked polyubiquitination, whereas ubiquitination with UbcH5 does not have any specificity of a lysine residue and the polyubiquitin chain remains linked to CHIP (Windheim et al., 2008). It has been shown in another finding that interaction with UbcH5 causes Lys^48^-linked ubiquitination and it leads to degradation of proteins (Murata et al., 2003; Schulman and Chen, 2005). An *in vitro* study has shown that CHIP, with the aid of UbcH5 and UbcH6, can efficiently ubiquitinate interferon regulatory factor 1 (IRF1), giving an idea of its modulation, under stress conditions (Narayan et al., 2011). Similarly, another study based on CHIP U-box domain showed that eight E2’s (Ube2D1, Ube2D2, Ube2D3, Ube2E1, Ube2E2, Ube2E3, Ube2N and Ube2W) can specifically interact with this domain (Soss et al., 2011). As evident from the above studies, CHIP interacts and cooperates with different E2’s. However, the basis of interaction towards a particular E2, thereby, producing different ubiquitin signals, is highly complex and still much work is needed to be done to understand this mechanism.

### CHIP, as a Co-Chaperone

**Figure 2:**
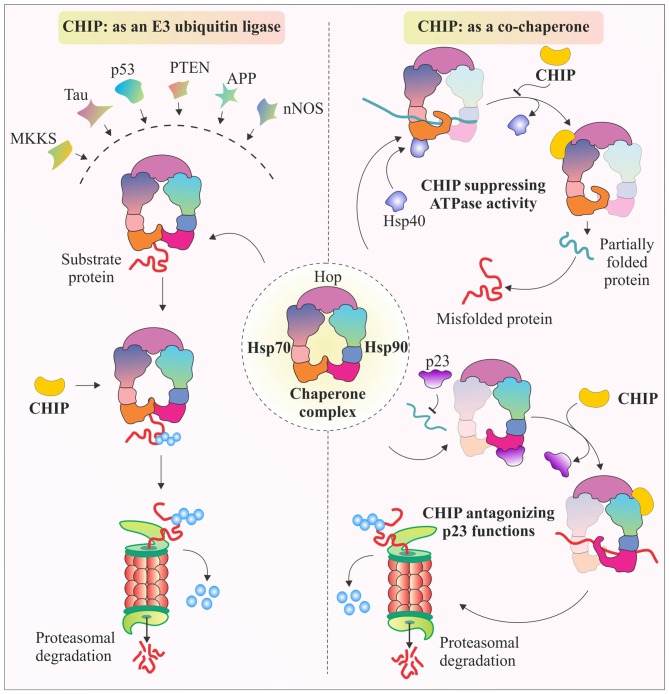
**Multifaceted CHIP, functions as E3 ubiquitin ligase and co-chaperone.** The illustration represents E3 ubiquitin ligase activity of CHIP through which, it targets major class of its client proteins, when they are bound to Hsp70-Hsp90 chaperone complex. It ubiquitinates these proteins to tag them for proteasomal degradation. CHIP is also known for its co-chaperone like functioning. The figure also represents antagonistic effects of CHIP against co-chaperones Hsp40 and p23. CHIP antagonizes Hsp40 by inhibiting its ATPase activity which helps Hsp70 to fold its substrates (McDonough and Patterson, 2003). Similarly, CHIP binds with Hsp90, causing release of p23, resulting in ubiquitination and further degradation of substrate proteins, such as glucocorticoid receptors (GR) and erythroblastic leukemia viral oncogene homolog 2 (ErbB2; Connell et al., 2001; Höhfeld et al., 2001; Xu et al., 2002; Theodoraki and Caplan, 2012).

There are various studies that provide evidence that CHIP tightly regulates chaperones. It not only assists in the functioning of chaperones, but also promotes or inhibits their co-chaperone modulated activities. One such example is the antagonistic regulation of Hop activity, by binding of CHIP to C-terminus of Hsp70 and Hsp90, using its TPR domain. Hop generally promotes folding of proteins in conjugation with Hsp70/90 complex, with the aid of Hsp40, stimulated ATPase activity of Hsc70. But, CHIP when binds with Hsp90 through its TPR chaperone adapter domain, further recruits E2 enzymes, ubiquitinate substrate proteins and marks them for proteasomal degradation (Ballinger et al., 1999; Höhfeld et al., 2001). As shown in Figure 2, CHIP competitively dissociates p23, another Hsp90 associated co-chaperone, which promotes Hsp90-substrate dissociation; whereas binding of CHIP stabilizes the complex and further ubiquitinates substrate protein for degradation. Similarly, CHIP can also regulate the functionality of Hsp70 by antagonizing the effect of Hsp40 in helping the Hsp70 associated polypeptides to fold. CHIP binding inhibits substrate folding-refolding pathway directed by Hsc70/Hsp70 chaperones (Höhfeld et al., 2001; McDonough and Patterson, 2003). Binding of CHIP with chaperones has been seen as a mechanism of modulation of the activities of the major chaperones in combination with other well known co-chaperone proteins. Such kind of regulatory roles of CHIP over molecular chaperones gives it an extra advantage of regulating levels of various substrate proteins, apart from its regular E3 ligase like functions in degradation of proteins.

### CHIP, as an E3 Ubiquitin Ligase

Similar to RING domain E3 ubiquitin ligases, which have a zinc binding motif, U-box domain containing E3 ubiquitin ligases use salt bridges and hydrogen bonds to stabilize their three-dimensional structures and perform their functions (Aravind and Koonin, 2000). The presence of U-box domain in CHIP led scientists to identify its potential as an E3 ubiquitin ligase enzyme (Hatakeyama et al., 2001). Till now, multiple evidence have been established and identified a wide and diverse array of substrates, which have been shown to be associated with CHIP with or without chaperones (Dickey et al., 2007; Faresse et al., 2010). These substrates then get ubiquitinated and degraded by either proteasomal or lysosomal pathways, depending upon their mode of ubiquitination, as described in detail in the next section. Interestingly, CHIP not only regulates numerous substrates, but also ubiquitinate chaperones, like Hsp70 and Hsp90 for their proteasomal degradation. As shown in Figure 2, major class of CHIP substrates are Hsp70-Hsp90 bound proteins, which are later ubiquitinated and degraded by E3 ligase activity of CHIP. Previously, it has been observed that after the depletion of substrates, CHIP may also ubiquitinate and degrade Hsp70 in a proteasome-dependent manner (Murata et al., 2001; Qian et al., 2006; Dickey et al., 2007; Kundrat and Regan, 2010).This versatile capability of CHIP to ubiquitinate in an array of patterns makes this protein unique with a master control over a large variety of substrates.

### E4 Like Activity of CHIP

Yeast protein UFD2 was first identified for its unique activity of binding with the ubiquitinated substrates and extends its ubiquitin chain in conjugation with other UPS components to make those substrates eligible for proteasomal degradation (Koegl et al., 1999). U-box proteins were not only identified as the third family of E3 ubiquitin ligases after HECT and RING finger families, but also reflect their activity as E4 enzymes (Hatakeyama et al., 2001). Among various E3 ubiquitin ligase enzymes, CHIP was the first to be identified for its E4 activity, in association with Parkin for Parkin-Associated Endothelin Receptor-Like Receptor (Pael-R) degradation (Imai et al., 2002). This activity makes it capable of adding further ubiquitin molecules, to form a polyubiquitin chain. It also shows the collaborative ability of CHIP with other E3 ubiquitin ligases in degradation of substrates (Hoppe, 2005). CHIP enhances the efficiency of Parkin to polyubiquitinate the Pael-R, resulting in its degradation via the proteasome. CHIP associates with Hsp70 to make Parkin free. As a result of this Parkin-Hsp70 dissociation, the activity of E2 ubiquitin-conjugating enzymes increases, which enhances the clearance of Pael-R, by Parkin from the cell (Imai et al., 2002).

## Chip: A Quality Controller of Cellular Proteome

**Figure 3:**
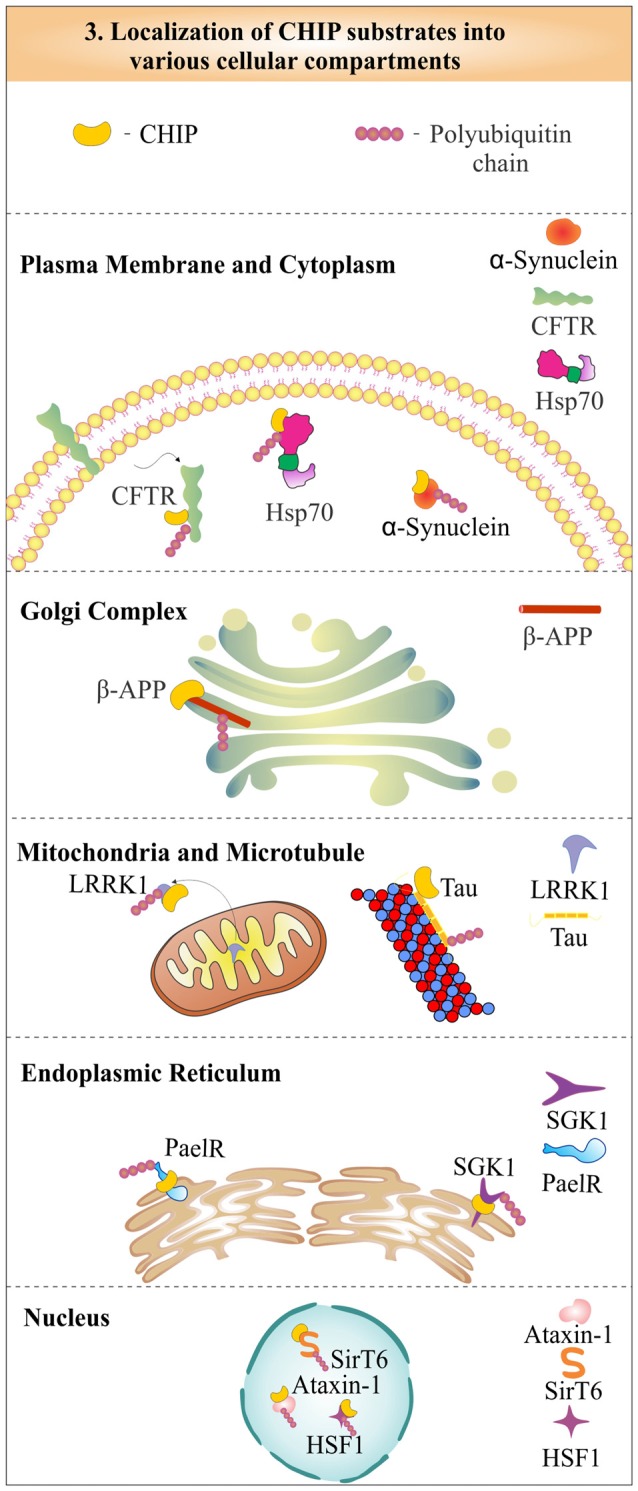
**Represenation of subcellular localization of CHIP in various cellular compartments.** CHIP is widely distributed in most of the subcellular compartments, including nucleus, cytoplasm, endoplasmic reticulum and plasma membrane, in association with its substrates.

CHIP was first identified as a cytoplasmic protein, but in later years, its functional role in the nucleus was also well established (Ballinger et al., 1999). The localization studies of CHIP with cystic-fibrosis transmembrane-conductance regulator (CFTR) proposed its colocalization at the endoplasmic reticulum membrane, to degrade it by the proteasome (Meacham et al., 2001). The role of CHIP in the cytoplasmic quality control system, later confirms its localization in the cytoplasm (Dai et al., 2005). It was found that CHIP is partly localized in the nucleus also, to perform functions like activation of heat shock factor 1 (HSF1; Dai et al., 2003). The cytoplasmic CHIP does not only take care of cytoplasmic proteins, but is also involved in the ubiquitination and degradation of mitochondrial proteins like leucine-rich repeat kinase 2 (LRRK2), a mutation in which has largely been implicated in the onset of many cases of familial Parkinson’s disease (Ding and Goldberg, 2009). CHIP also ubiquitinates and degrades substrates like microtubule-associated tau and β-amyloid precursor protein (β-APP), which is localized in the Golgi complex (Kumar et al., 2007; Wang and Mandelkow, 2012). Hence, enormous data presented by various studies showed that function of CHIP is not confined to any special cellular location, but, it plays significant roles in the regulation of a variety of substrates, present in different cellular locations and compartments. In Figure 3, we depict the subcellular distribution of CHIP with few of its substrate proteins to evince its cellular localization and functional potency. As mentioned earlier, CHIP is involved in both pathways of protein quality control: UPS and autophagy. The subsections below describe and categorize substrates of CHIP, in accordance with their degradative mechanisms. Most of its substrates have found to be a proteasomal target, whereas some of them are degraded through the lysosomal pathway. Interestingly, few of them *viz*. inositol-requiring enzyme 1 (IRE1) are only modified functionally, instead of being degraded by any of these degradation pathways (Zhu et al., 2014).

### Ubiquitin Proteasome System and CHIP Harmony for Regulating Cellular Functions Under Stress Conditions

To describe the involvement of CHIP in the UPS, it is important to explain how different substrates are degraded by using proteasomal machinery. The list of CHIP substrates begins with heat shock protein Hsp70, which is not only functionally regulated by CHIP, but is also degraded through the proteasomal pathway by its E3 ubiquitin ligase activity (Qian et al., 2006). CHIP efficiency of ubiquitination and proteasomal degradation is higher for Hsp70 than Hsc70, although, these two are highly similar in structure and homologous in sequence. There is also a difference in the distribution of multiple ubiquitin chain formation by CHIP among these two proteins (Soss et al., 2015). Recently, studies on lens epithelial cells reported that knockdown of CHIP affects the Hsp70 level during heat stress, the concentration of Hsp70 in those cells is less, as compared to negative control cells (Zhang et al., 2015). The mouse model of ischemia/reperfusion-induced heart injury also suggested that CHIP suppresses Hsp70 in the heart (Zhao et al., 2012). Molecular chaperone Hsp70, in complex with CHIP, leads to ubiquitination and elimination of various cellular proteins, whereas Hsp90 and their client protein complexes need to be destabilized first and then their ubiquitination and removal takes place (Pratt et al., 2015). It was further illustrated by a study, in which CHIP along with Hsp90 controls the stabilization and degradation of proteins such as aryl hydrocarbon receptor (AhR) and SUMO/sentrin protease 3 (SENP3), which is involved in cellular response to mild oxidative stress (Morales and Perdew, 2007; Yan et al., 2010). The optimal levels of DNA polymerase β, DNA ligase III and X-ray repair cross-complementing group-1 (XRCC1), the base excision repair enzymes, are maintained by proteasomal degradation, through CHIP (Parsons et al., 2008; Sobol, 2008). All these examples show the cytoprotective nature of CHIP under various stress conditions including heat shock, oxidative stress and DNA damage etc.

#### Molecular Functions of CHIP in the Regulation of Signaling Pathways and Cellular Proliferation

The proteins, which are regulated by CHIP, are shown to be involved in many signaling pathways, and thus a dysregulation may finally lead to a condition of cancer, neurodegeneration, and other stress-related pathologies (Shang et al., 2014). The signaling kinases like AKR/J thyoma protein kinase (Akt), which is responsible for phosphorylation of tau and protein tyrosine kinase 6 (PTK6), that is highly expressed in various cancers are degraded by CHIP (Dickey et al., 2008; Kang et al., 2012). Regulation of signaling by CHIP was also associated with degradation of a typical protein kinase C ζ (PKCζ) and sarcoma viral oncogene homolog (Src), involved in Toll-like receptor-mediated immunity (Yang et al., 2011). Other kinases, like nucleophosmin-anaplastic lymphoma kinase (NPM-ALK) and liver kinase B1 (LKB1) are degraded by E3 ubiquitin ligase activity of CHIP (Bonvini et al., 2004; Gaude et al., 2012). Nuclear factor-kB (NF-kB), TNF receptor-associated factor 2 (TRAF2) and Caspase recruitment domain (CAD) containing membrane-associated guanylate kinase (CARMA1), key molecules participating in the regulation of the NF-kB pathway is also regulated by ubiquitination and degradation through CHIP (Jang et al., 2011; Wang et al., 2013a). The studies also found the role of CHIP in maintaining the cellular concentrations of proteins such as MAPK/ERK kinase kinase 2 (MEKK2) and mixed-linkage kinase (MLK3), which are shown to be involved in important signaling pathways, like extracellular signal-regulated kinase (ERK), mitogen-activated protein kinase (MAPK) and c-Jun N-terminal kinase (JNK) pathways, which regulate migration, invasion, proliferation and apoptosis-like important cellular phenomenon (Maruyama et al., 2010; Blessing et al., 2014). It maintains a steady level of kinases such as dual leucine zipper-bearing kinase (DLK) and serum and glucocorticoid regulated kinase-1 (SGK-1) in the cell, in complex with Hsp70 (Belova et al., 2006; Daviau et al., 2006). Hsp90 client protein, migration inhibitory factor (MIF), which is involved in many signaling pathways, is also a well-known substrate of CHIP (Schulz et al., 2012). Breakpoint cluster abelson murine leukemia viral oncogene (BCR-ABL) and C-terminal binding protein 2 (CtBP2) are other two proteins, added in this list of CHIP substrates (Tsukahara and Maru, 2010; Lee and Yoo, 2013).

The regulation of turnover of various receptors, that are involved in signaling processes, by CHIP, is performed by degrading proteins like glucocorticoid receptors (GR), estrogen alpha receptor (ERα) and androgen receptor (AR), using UPS (Wang and DeFranco, 2005; Zhang et al., 2011; Sarkar et al., 2014). The E3 ubiquitin ligase function of CHIP maintains the cellular level of recepteur d’origine nantais kinase (Ron) tyrosine kinase receptor that binds with ligand macrophage-stimulating protein to give the signal for proliferation and migration of cells (Germano et al., 2006). Another protein of phosphoinositide 3-kinase (PI3K) signaling pathway, phosphatase and tensin homolog deleted on chromosome TEN (PTEN) interacts with the TPR domain of CHIP, by parts of its N-terminal domain for ubiquitination and degradation (Ahmed et al., 2012). It also regulates human telomerase reverse transcriptase (hTERT) and hence modulates telomerase activity in cancer cells (Lee et al., 2010). A tumor suppressor gene, menin, mutation in which causes multiple endocrine neoplasia type1, is rapidly degraded after being ubiquitinated by CHIP (Yaguchi et al., 2004).

CHIP is also important for the regulation of many proteins involved in the formation of tumors, known as proto-oncogenes, for example cErbB2/Neu and c-Myc (Xu et al., 2002; Paul et al., 2013). Receptor tyrosine kinase AXL, that binds to the growth arrest-specific protein 6, regulates the progression of many cancers, is also a substrate of CHIP (Krishnamoorthy et al., 2013). Overexpression of CHIP maintains the critical cellular levels of substrate proteins and inhibits their over-accumulation, such as erythroblastic leukemia viral oncogene homolog 2 (ErbB2), a member of the receptor tyrosine kinase family that control normal cell proliferation, differentiation and migration. The new emerging anti-cancer strategies, involving downregulation of ErbB2, also focus on the CHIP-mediated degradation of this protein in association with molecular chaperones (Zhou et al., 2003). The mutant p53, another tumor-causing protein, is a substrate of CHIP and chaperone complex (Esser et al., 2005). Tumor cell migration regulating protein, profilin, is also a notable substrate of CHIP (Choi et al., 2014). As apoptosis signal-regulating kinase 1 (ASK1) contain TPR acceptor site, it was found that CHIP, with its TPR domain binds with this site to ubiquitinate and degrade it by the proteasome (Hwang et al., 2005). A truncated form of a mitochondrial protein, apoptosis inducing factor (tAIF) is ubiquitinated and degraded by CHIP in Hsp70 dependent manner (Oh et al., 2011). A recent study summarizes that this interaction is disrupted by cyclin-dependent kinase (Cdk5), which phosphorylates CHIP at Ser20 position. Even after this disruption, E3 ubiquitin ligase activity of CHIP remains intact, but this modification causes inhibition of tAIF proteasomal degradation and thus leads to the death of neurons (Kim et al., 2016). Endonuclease G, another mitochondrial protein, which induces apoptosis is ubiquitinated and degraded by CHIP to protect cells from cell death, induced by oxidative stress (Lee et al., 2013). Recently, it was also found that protein arginine methyltransferase 5 (PRMT5), an oncoprotein, expressing at high concentration in a number of human cancers, is negatively regulated by CHIP, which ubiquitinates and degrade PRMT5 via proteasomal degradation (Zhang et al., 2016). These line of evidence showing a diverse range of substrates, including signaling molecules, receptor proteins, various kinases, a number of oncoproteins and tumor suppressors make this protein a potential target, which, in future, may be exploited for the development of anti-cancer strategies.

#### Cellular Functions and Relations of CHIP Associated with Other Crucial Molecular Physiological Partners

Enzymes are also in the list of CHIP substrates, such as Hsp90 linked guanylyl cyclate (GUCY) and neuronal enzymes like neuronal nitric oxide synthase (nNOS; Peng et al., 2004; Xia et al., 2007). GTP cyclohydrolase I degradation controlled by CHIP, regulates pulmonary blood flow (Sun et al., 2011). Proteins like cytochrome 450 isoform 3A4 (CYP3A4) and NAD(P)H: quinone oxidoreductase 1 (NQO1), are also governed by CHIP for their cellular levels (Tsvetkov et al., 2011; Wang et al., 2012). The complex of CHIP/Hsp70 involves association of many other co-chaperones such as Hsp40, BAG-1 and BAG-3. These co-chaperones, especially BAG-1, facilitate the ubiquitin tagging of the substrate proteins, like rapidly accelerated fibrosarcoma 1 (Raf1), and mark them for 26S proteasomal destruction. BAG-1 interacts with CHIP and helps in the transfer of the substrate from Hsp70 to CHIP-mediated proteasomal degradation (Demand et al., 2001). Hsp70 bound CHIP was also observed to take part in balancing critical concentrations of proteins, such as mutant keratins and hypoxia-inducible factor-1α (HIF-1α), which is a transcription factor for proteins which are up-regulated in response to low oxygen levels (Luo et al., 2010; Löffek et al., 2010). Thus, CHIP also controls adaptive responses of cells in low oxygen conditions. Other studies also suggest the involvement of CHIP in regulating the levels of other Hsp90 client proteins such as runt-related transcription factor 1 (Runx1) and LRRK2, via UPS, a protein whose mutation is linked to Parkinson’s disease (Ding and Goldberg, 2009; Shang et al., 2009). Hsp90 inhibitor molecules are investigated as it has shown to control important signaling pathways, which are up-regulated in many diseases. Thus, controlling the substrate accumulation of Hsp90 client proteins by overexpressing CHIP can provide options for treatment of various pathologies (Pratt et al., 2015). The roles of CHIP/Hsp70 in regulation and selective removal of aggregated protein inclusions from the cell in various neurodegenerative disorders cannot be neglected. A detailed picture of its involvement in various neurological disorders has been presented in the later section of this review.

Other anomalies of the genetic disorders such as McKusick-Kaufman syndrome (MKKS), includes removal of mutant MKKS protein from the cell by CHIP (Hirayama et al., 2008). Translation-transcription regulators like eukaryotic translation initiation factor 4E (eIF4E), a forkhead transcription factor 1 (FoxO1) and histone deacetylase 6 (HDAC6) are also ubiquitinated and degraded by proteasomal degradation via CHIP interaction (Murata and Shimotohno, 2006; Li et al., 2009; Cook et al., 2012). The E3 ubiquitin ligase CHIP is also responsible for degradation of myocardin and katanin p60, hence, affects the differentiation in smooth muscle cells and axonal growth, respectively (Xie et al., 2009; Yang et al., 2013). The cardioprotective effect of CHIP is also because of ubiquitination and degradation of inducible cyclic AMP early repressor (ICER; Woo et al., 2010). Geldanamycin (Hsp90 inhibitor) induces the degradation of estrogen receptor alpha; CHIP not only increases this effect, but also maintains the basal level of this receptor (Fan et al., 2005). Sma-mother against decapentaplegic 1 (Smad1) and Smad4 are both coexpressed with the CHIP, and it was found that Smad1 is preferentially degraded by the proteasome to block bone morphogenetic proteins (BMP) signal transduction (Li et al., 2007). In the case of interferon regulating factor (IRF-1), trichostatin A treatment to cells causes enhanced proteasomal degradation of IRF-1, by association with CHIP (Gao et al., 2013). CHIP interaction with Hsp70 also controls the critical level of neuronal nicotinic acetylcholine receptors (nAChRs), which are involved in ganglionic transmission and take part in smoking-related diseases (Teng et al., 2015). Interaction of CHIP and Hsp70-Hsp90 chaperones largely regulate a broad range of proteins, which are part of crucial and important regulatory pathways. Regulatory control over transcriptional and translational regulators designates CHIP as a master controller of such a large network of proteins which play critical roles in cellular metabolism, signaling and proliferation.

### CHIP-Mediated Autophagic Degradation with the Help of Chaperones

**Figure 4:**
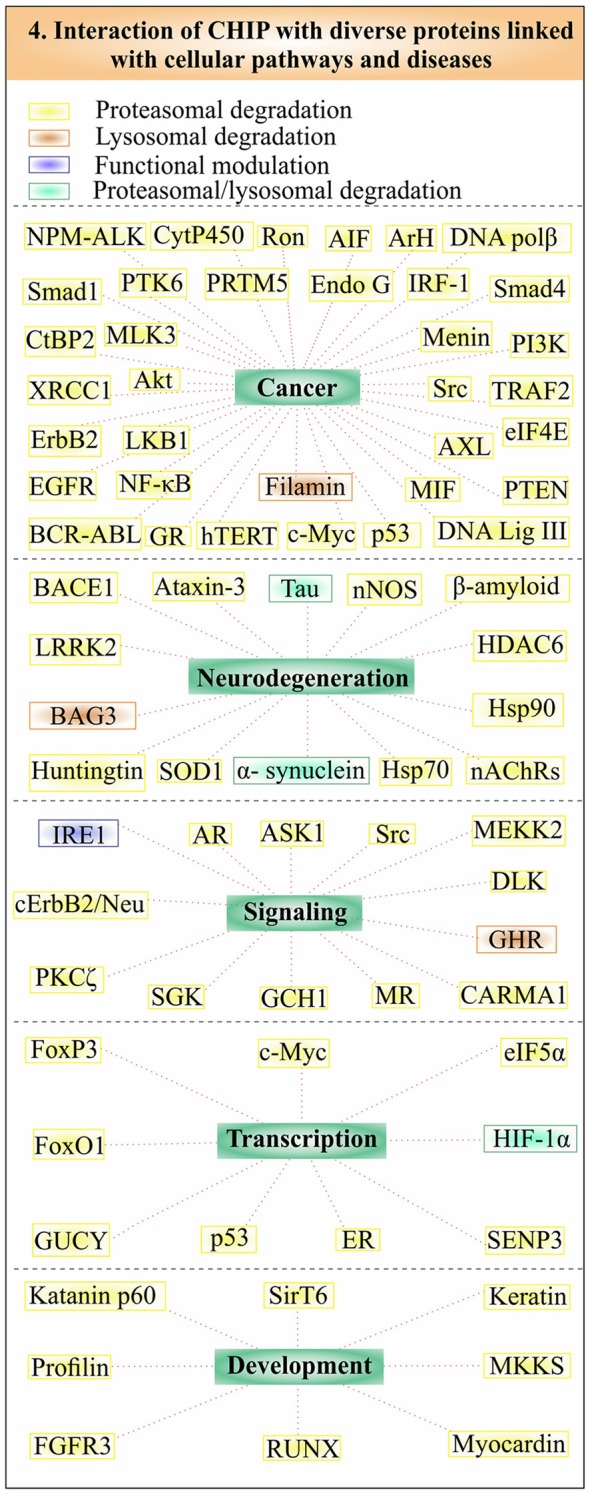
**CHIP linked substrates in multiple cellular pathways and abnormalities.** CHIP substrates form a large group of proteins, implicated in various important pathways and pathologies. Substrates are subdivided in accordance with their association with cancer, neurodegeneration, signaling, transcriptional regulation and development. Substrates have been put into different boxes according to their degradative pathways. Yellow boxes represent substrates, which are degraded by the proteasome, whereas brown boxes represent the lysosomal degradation of CHIP substrates. Substrates degraded by both proteasomal and lysosomal pathways are shown in the green box. CHIP also ubiquitinates few of its substrates to functionally regulate them instead of degrading, shown within the purple box.

Studies found that CHIP also plays a crucial role in maintaining cellular level of many crucial proteins, by using the degradation pathway of autophagy. It has been observed that CHIP/Hsp70 complex alleviates cells from the proteotoxic stress generated by phosphorylated tau and mutant alpha-synuclein by using both the systems of UPS and autophagy (Shin et al., 2005; Wang and Mandelkow, 2012; Chesser et al., 2013). Regulation of HIF1 by CHIP also involves oxygen dependent proteasomal degradation and CMA (Luo et al., 2010; Ferreira et al., 2013). Various other regulatory proteins and co-chaperones are found to be associated with CHIP/Hsp70 complexes to reduce the proteotoxic load of the cell by using autophagy, one such protein is growth hormone receptor (GHR). Endocytosis of GHR is controlled by mainly two types of modifications: phosphorylation and ubiquitination by CHIP (Slotman et al., 2012; Sedek et al., 2014). A coordination of CHIP and Hsp70 with molecules such as BAG3, heat shock protein B8 (HspB8), directly control client proteins using the machinery of autophagy, a process known as CASA (Gamerdinger et al., 2011). Filamin, the Z-disk maintaining protein is ubiquitinylated and degraded by CHIP through CASA, so that actin can anchor properly in striated muscles (Arndt et al., 2010). This mechanism helps to maintain the integration of *Z* line and prevents muscle weakness. Other studies suggest HspB8, BAG3 and CHIP/Hsp70 complex effectively causes solubilization and thus eliminates aggregates of mutant superoxide dismutase (SOD1) via the mechanism of autophagy. The autophagic flux of many substrates in the cell is controlled by CHIP, as observed in CHIP knockdown cells, where it regulates autophagic marker proteins such as p62 and microtubule-associated protein 1A/1B-light chain 3 (LC3; Guo et al., 2015). In Figure 4, we tried to categorize the substrates of CHIP in accordance with pathways, through which they are degraded and diseases, where these proteins are involved. The ability of CHIP to degrade its substrate proteins by multiple quality control pathways, make its presence crucial for maintainance of cellular proteome balance.

## Chip, A Molecule Associated with Multiple Neurodegenerative Diseases

In the above section, we have described the role of CHIP in quality control pathways, to maintain healthy cellular proteome. It is evident from earlier research works, done by different groups that most of the neurodegenerative diseases start developing with few common pathological hallmarks (Jellinger, 2001). Extra- or intracellular stresses and simultaneously, insufficient functioning of various arms of cellular protein quality control machinery may lead to structural modifications of some class of cellular proteins, which may form misfolded oligomers. In many cases, they start accumulating and forming inclusion bodies by entangling with other functional cellular proteins, as evident from microscopic analyses of different diseased brain tissues (Irvine et al., 2008). Such kinds of toxic proteinaceous species may lead to an array of neurological disorders e.g., Alzheimer’s disease, Parkinson’s disease, Huntington’s disease, Amyotrophic lateral sclerosis (ALS) and Lafora disease, etc. (Dickey et al., 2007).

**Figure 5:**
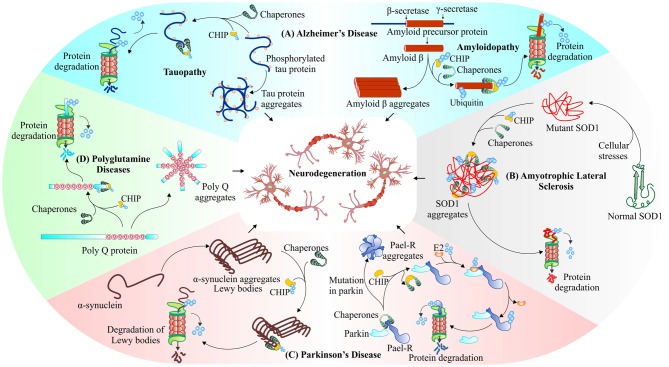
**CHIP, a major E3 ubiquitin ligase of neurodegenerative disorders.** CHIP ameliorates proteotoxicities in various proteinopathies. Studies have shown that CHIP targets phosphorylated tau (Petrucelli et al., 2004; Qian et al., 2006; Koren et al., 2009), beta-amyloid (Kumar et al., 2012) in Alzheimer’s disease **(A)**, mutant superoxide dismutase (SOD1) aggregates (Urushitani et al., 2004) in Amyotrophic lateral sclerosis (ALS; **B)**, Parkin-Associated Endothelin Receptor-Like Receptor (Pael-R; Imai et al., 2002; McDonough and Patterson, 2003; Kumar et al., 2012), alpha-synuclein (Shin et al., 2005) in Parkinson’s disease **(C)** and polyglutamine protein aggregates (Jana et al., 2005; Williams et al., 2009) in polyglutamine diseases **(D)**, for their proteasomal degradation.

An interesting study in model organisms for identification of common genetic modifiers of neurodegenerative diseases has also shown the importance of CHIP (Chen et al., 2012). The gene expression analysis data of SAMP8 mouse model of Alzheimer’s disease by cDNA microarray technique, form a protein network where CHIP represents an important node, which signifies its influence on the disease (Cheng et al., 2013). In Figure 5, we summarized the roles of CHIP in different neurodegenerative disorders, where we have shown its association with the misfolded proteins. Here, in this section of the review, we are focusing on various roles attributed to E3 ubiquitin ligase like activities of CHIP, in various neurodegenerative diseases.

### Alzheimer’s Disease

The evidence points towards two major hypotheses as causative factors for this severe neurodegenerative disease. The disease begins with the formation of intracellular neurofibrillary tangles of phosphorylated tau, a modified form of microtubule-associated tau protein, as the major cause of Alzheimer’s (Binder et al., 2005). *In vivo* studies clearly suggests that in conjugation with UbcH5B, an E2 enzyme, CHIP-Hsp70 complex polyubiquitylates phosphorylated form of tau in both K48 as well as K63 linked manners and directs it for proteasomal or autophagic degradation respectively (Petrucelli et al., 2004). A second hypothesis, explaining the processing of amyloid precursor protein (APP) by β-secretase, leads to accumulation of amyloid beta (Aβ42) peptide fragments and then formation of extra-cellular amyloid plaques, causing neuronal loss of function or synaptic loss, and in later stages loss of neurons may lead to death (Adalbert et al., 2007). Previous studies showed that colocalization of CHIP with APP stabilizes its normal cellular form; the same study also identified the involvement of CHIP in conjugation with Hsp70 and Hsp90, in suppressing accumulation of toxic Aβ42 peptide (Kumar et al., 2007). CHIP, therefore, enhances overall cellular health, with increased rate of cell survival (Shimura et al., 2004; Chesser et al., 2013). In Figure 5A, we have shown the interaction of CHIP with both these toxic proteins and their removal through the proteasomal pathway. The phosphorylated tau and β-amyloid interact with CHIP. This interaction further leads to ubiquitination and proteasomal degradation of these proteins.

### Amyotrophic Lateral Sclerosis

The other well-known neurodegenerative disorder, ALS, which occurs due to mutations in SOD1, an antioxidant enzyme, present on mitochondrial membranes and TAR DNA-binding protein-43 (TDP-43), includes the roles of E3 ubiquitin ligase CHIP. It mediates polyubiquitination of Hsp70/Hsc70, which preferentially promotes degradation of SOD1 via 26S proteasome, as shown in Figure 5B (Urushitani et al., 2004). The overexpression of CHIP decreases cytotoxicity in cultured human cells by reducing levels of mutant SOD1 together with Hsp70/Hsc70 and Hsp90 (Choi and Lee, 2010). The degradation of mutant SOD1 protein through autophagy is carried out by Bag3/HspB8/Hsc70/CHIP complex that inserts mutant protein into autophagosome (Crippa et al., 2010). Another study describes increasing efficiency of Dorfin ligase by the development of Dorfin-CHIP fusion protein for degradation of mutant SOD1 protein (Ishigaki et al., 2007). Further research on the involvement of CHIP in targeting proteinaceous aggregates apart from SOD1, which is involved in ALS, will contribute in identifying its potential roles in ameliorating such kind of proteinopathies. TDP-43, cytoplasmic inclusions are another hallmark of ALS. These aggregates of TDP-43 interact with HDAC6, a substrate of CHIP/Hsp70. The complex of HDAC6/CHIP/Hsp70, in normal conditions, has the capability of deacetylating TDP-43, but they fail in case of oxidative stimuli of high potency (Cohen et al., 2015).

### Parkinson’s Disease

Parkinson’s disease also involves the formation of various proteotoxic inclusions inside the neurons, which may later result in dementia or in extreme cases, the death of patients (Irvine et al., 2008). The inclusions forming proteins of this disease are Pael-R, α-synuclein and LRRK2. Pael-R, a G-protein coupled receptor when accumulates in endoplasmic reticulum causes ER-stress that leads to neuronal death by an unexplored mechanism (Uversky and Fink, 2007; Ko et al., 2009). CHIP mediates the dissociation of Hsp70 and Parkin, a known E3 ubiquitin ligase of Pael-R, increases the ubiquitination of Pael-R and subsequent degradation by UPS (Imai et al., 2002). Lewy bodies are the cytoplasmic fibrillar aggregates of α-synuclein, known as a pathological hallmark of Parkinson’s disease (Irvine et al., 2008). Overexpression of CHIP in cell culture models showed its colocalization with α-synuclein and Hsp70 and a reduction in levels of α-synuclein associated inclusion bodies; it also caused alteration in the morphology of these inclusions. The direct interaction of CHIP and α-synuclein shows that clearance of α-synuclein is not only carried out by proteasomal degradation, but is also mediated by lysosomal pathways (Shin et al., 2005).

The oligomeric form of α-synuclein is preferentially recognized by CHIP for degradation in the TPR domain dependent and U-box independent manner (Tetzlaff et al., 2008). In further studies, it was observed that the ubiquitination of α-synuclein is regulated by BCL-2-associated athanogene5 (BAG5), it changes CHIP ability of reducing α-synuclein oligomerization (Kalia et al., 2011). As shown in Figure 5C, the above two described Parkinson’s linked protein Pael-R and α-synuclein are degraded by CHIP-mediated ubiquitination. A mutation in mitochondria-associated LRRK2 protein is found to be involved in the onset of familial Parkinson’s disease have also been reported as a substrate of CHIP (Ding and Goldberg, 2009; Ko et al., 2009). Another important role, this E3 plays, is to maintain the mitochondrial integrity, its homeostasis and overall functional health. CHIP knockout mice have impaired mitochondria, showing defective mitophagy, malfunctioned antioxidant defense and increased overall protein oxidation with elevated levels of mitochondrial stress related proteins. Increase in cellular protein and lipid oxidation are the major causes of motor impairments; deficiency of CHIP generates an oxidative insult which may lead to a decrease in neuronal survival (Palubinsky et al., 2015).

### Polyglutamine Diseases

Polyglutamine diseases like Huntington’s disease and Spinocerebellar ataxia, also have involvement of CHIP substrate proteins. Co-immunoprecipitation studies of CHIP and polyglutamine-expanded proteins, huntingtin and ataxin-3 demonstrated CHIP’s role in polyglutamine diseases. E3 ubiquitin ligase CHIP along with Hsp70 ubiquitinates and degrades polyglutamine aggregates of mutant huntingtin and ataxin-3 (Jana et al., 2005; Dikshit and Jana, 2007), as depicted in Figure 5D. In further studies, Huntington’s disease cell models, primary neurons and zebra fish models show a reduction in aggregates by the co-chaperone activity of CHIP. Also, CHIP haploinsufficient mouse models showed an accelerated disease phenotype giving an idea of the protective nature of CHIP (Miller et al., 2005). Similarly, in fly-model of Spinocerebellar ataxia, with wild type and polyglutamine expanded ataxin-1, CHIP was shown to reduce toxicity (Al-Ramahi et al., 2006). CHIP ubiquitinates and degrades ataxin-1, decreases its solubility, that causes the formation of insoluble aggregates, which sequesters other misfolded proteins to provide cellular protection (Choi et al., 2007). Mice with spinal and bulbar muscular atrophy, a polyglutamine expansion disease, showed improvement on overexpression of CHIP by reducing both wild-type and poly Q-expanded androgenic receptor (Adachi et al., 2007).

### Lafora Disease

An autosomal recessive disorder involving neurodegeneration, lafora disease, is a result of a mutation in either substrate laforin or its E3 ubiquitin ligase malin (Gentry et al., 2005). Lafora bodies, in this disease, are polyglucosan inclusions. CHIP associates with malin-laforin complex and translocate the same to the nucleus, resulting in the activation of cellular response through upregulation of HSF1 (Sengupta et al., 2011). Malin, which is involved in the degradation of laforin, itself, shows the tendency of aggregate formation. Mutants of malin are not stable like wild type malin, and form cytoplasmic and nuclear aggregates; co-chaperone CHIP modulates stability of malin by altering Hsp70 activity (Rao et al., 2010).

## Roles of Chip in Development and Aging Processes

CHIP is an E3 ubiquitin ligase that has the capability to regulate its substrate proteins, right from their folding, and up to the stages of its degradation inside cells. It associates with different chaperone complexes to monitor the folding processes, as well as different protein degradation pathways, such as proteasome and autophagy. It presents a direct connecting link between these different arms of the protein quality control system: molecular chaperones, UPS, and autophagy, as described in the above sections. Apart from playing crucial roles in protein structural modifications by its co-chaperone activity, CHIP also takes part in the development of organisms. We, here, try to emphasize few crucial roles of this E3 ubiquitin ligase in development processes. CHIP substrate Runx2 is involved in regulation of osteoblast differentiation. CHIP co-chaperone domain TPR and C-terminus of Runx2 interacts with each other and results in ubiquitination and degradation of Runx2, which suppresses differentiation of preosteoblasts into osteoblasts. It could probably be due to the repressed transcriptional activity of Runx2. On the other hand, this inhibition of Runx2 by CHIP results in increased rate of adipogenesis (Li X. et al., 2008).

Another study showed the negative effect of the CHIP knockout on total body weight of mice. The study points out its role in the overall aging process of the organism. CHIP knockout resulted in increased rate of atrophy, which was measured by decreased size of the thymus, testis and skeletal muscles and the cardiac hypertrophy also signifies the increased rate of aging. Histological considerations were also taken into account, apart from these anatomical studies. It was found that CHIP knockout has a universal effect on the dermis, resulting in thinner dermis layers as compared to control, as well as the subcutaneous deposits of fat, which was also reduced significantly. Bone mineral density was also decreased with symptoms of kyphosis (Min et al., 2008). CHIP has also been found to be involved in early neuronal development stages. The study conducted by Yang et al. (2013) shows that CHIP regulates the level of katanin-p60 protein, which is required for the proper axonal growth in early developmental stages. CHIP antagonizes the effects of a deubiquitylating enzyme, USP47, by ubiquitinating and destabilizing katanin-p60, and hence presents an important role in axonal growth (Yang et al., 2013). Another study showed abnormal motor disturbances with altered phenotypic and behavioral functions in CHIP heterozygous mice (McLaughlin et al., 2012). CHIP may also have some indirect regulatory roles over various neurodevelopmental processes through its large number of substrates, where it regulates various proteins which are directly involved in brain development processes. For example, one of its substrates, PTEN is a key signaling molecule that have direct role in various developmental processes like neuronal differentiation and neurite growth (Li et al., 2003; Hsia et al., 2014). The cellular level of PTEN is largely regulated by its proteasomal degradation, governed by various E3 ubiquitin ligases, including CHIP (Ahmed et al., 2012). Cytoskeleton of large neurons are majorly built by cytoskeletal proteins, neurofilaments. Evidence shows that CHIP has direct involvement in proteasomal degradation of neurofilaments medium subunit of 95 kDa, which are highly damaged and decreased in the less understood mechanism of 2, 5-hexanedione mediated neuropathology. Therefore, involvement of CHIP-mediated proteasomal degradation presents a regulatory mechanism to maintain the levels of neurofilament proteins (Wang Q. et al., 2011; Dale and Garcia, 2012). Although these studies indicate possibilities of CHIP involvement in various developmental processes; a lack of function of which may result in severe developmental abnormalities. These reports give us some clues to further investigate its roles in more details.

Involvement of CHIP in the regulation of aging processes is also very crucial and yet to be established completely. Apart from activation of HSF1, CHIP also affects the activity and stability of the sirtuin family of proteins, which importantly regulate DNA repair mechanisms and maintain cellular homeostasis, by providing required genomic integrity. It was observed that in mouse embryonic fibroblast and human embryonic kidney cells, CHIP knockdown causes post-translational decay of Sirtuin (SirT6) protein levels in the cells. Ubiquitination of SirT6 at K170 position by CHIP E3 ubiquitin ligase stabilizes the protein, which is rather degraded by some unknown E3 ubiquitin ligase. Such kind of positive regulation of SirT6 by CHIP provides a novel example where these two arms of DNA and protein quality maintenance systems cooperatively regulate homeostasis and aging processes (Ronnebaum et al., 2013). Exome sequencing analysis in a family affected with ataxia, found the loss of function mutation in CHIP resulting in hypogonadism and ataxia like symptoms. The reported Thr346Met mutation resulted in Gordon Holmes Syndrome and other accelerated aging related changes which may probably be a result of protein instability or impaired substrate binding (Heimdal et al., 2014; Shi et al., 2014). Therefore, apart from development, aging is another major area of research, where detailed reports stating direct roles of CHIP are still missing, which gives us an opportunity to dig out further, the possibilities of CHIP being targeted as a candidate for anti-aging strategies.

## Chip: Implications in Various Diseases

Apart from the above described roles of CHIP in neurodegeneration, development and aging, strong evidence has indicated the importance of CHIP in diseases, such as cancer, cardiac, muscular disorders, etc. A study in past has elucidated the potential of CHIP as a biomarker and therapeutic target in various human cancers (Sun et al., 2014). The strategies have been adapted to target E1 enzymes, chaperones, histone deacetylases, proteasomal subunits and E3 ubiquitin ligases, like mouse double minute 2 homolog (MDM2), anaphase-promoting complex (APC), etc., to treat various types of cancers with varying success (Eldridge and O’brien, 2010; Shen et al., 2013; Weathington and Mallampalli, 2014). As in renal cell carcinoma and gastric cancer, decreased CHIP expression has shown potential to serve as a potent biomarker of angiogenesis (Wang et al., 2013b; Sun et al., 2014). Similarly, in esophageal squamous cell carcinoma patients, elevated CHIP expression levels in metastatic lymph node have shown a high prognostic value (Wen et al., 2013). CHIP’s ability to downregulate ErbB2 makes it a potential indicator for breast cancer patients’ survival (Jan et al., 2011). Besides being extensively studied for prognostic ability, CHIP has also been induced with the help of certain small molecules.

Trehalose, a disaccharide, improves the deteriorating changes taking place in patients with ataxia due to a genetic mutation in STUB1/CHIP. Trehalose, apart from activation of autophagy, also increases the levels of CHIP and Hsp70 (Casarejos et al., 2014). Another molecule quercetin, a flavonoid present in fruits and vegetables, is a chemopreventive agent in cancer; shows its beneficiary effects by down regulating ErbB2 and upregulating its E3 ubiquitin ligase CHIP (Jeong et al., 2008). Another interesting example of using small natural molecule is gambogic acid, a xanthonoid, obtained from tree *Garcinia hanburyi* that inhibits the interaction of Hsp90 and mutant p53 to make it available for CHIP-mediated proteasomal degradation in cancer cells (Wang J. et al., 2011). Similarly, curcumin has been shown to induce CHIP-ErbB2 interaction, thereby reducing ErbB2 levels, a strategy shown to have an enormous potential in controlling metastasis (Jung et al., 2007). Besides these, a recent study also showed a protective effect of CHIP against ER stress-mediated cell death in the central nervous system, by preventing upregulation of p53 pathway and regulating unfolded protein response pathways (Cabral Miranda et al., 2015). Also, upregulating CHIP activity with Hsp90 inhibitors like 17-N-allylamino-17-demethoxygeldanamycin, for anticancer therapy could be an effective strategy (Paul et al., 2013).

Deubiquitinases (DUBs) are special class of proteases which can release ubiquitin from substrate proteins by breaking the peptide bonds in between substrate and ubiquitin molecules. Regulating DUBs activities is assumed to have some possible therapeutic benefits. Ataxin-3, one of the most studied DUB, found to have a direct interaction with CHIP along with an E2 enzyme Ube2W in degradation of its substrates. An important mechanism of regulation of DUB ataxin-3 by its ubiquitination is also present inside the cells. CHIP has shown to have the capacity to ubiquitinate ataxin-3 and in turn may have a regulatory effect on this neuroprotective DUB enzyme (Scaglione et al., 2011; Tsou et al., 2013). A different therapeutic strategy was proposed to enhance the efficacy of anticancer drugs, through CHIP, by modulating CYP3A4 functioning (Peer et al., 2011). CHIP also has a pivotal role in cardiac disorders, where it provides cardioprotection by degrading inducible cAMP early repressor in complex with extracellular stress-regulated kinase 5 (Woo et al., 2010). All the above described roles of CHIP, in various disorders elucidate its significance for future research to develop advanced strategies to control these pathological disorders. Although, CHIP is a potential molecule for effectively targeting multiple cellular targets involved in several diseases, still success in devising any notable strategy to treat any of these diseases is not reported so far. So, a precise focus on some key questions, which are yet to be answered, must be given and more conceptual understanding of its functioning inside the cell should be developed.

## Fundamental Challenges and Unsolved Puzzles

Many years of research has established CHIP, as a unique molecule, which has a large number of interacting partners. An interaction network of such a wide variety of substrates enables this E3 ligase enzyme to take part, directly or indirectly, in an array of cellular functions including metabolism, signaling, proliferation and apoptosis. The structure of CHIP, consisting of both TPR and U-box domain, is also a crucial factor in deciding its functional capabilities. Performing the roles of co-chaperone and E3 ligase simultaneously needs enormous molecular capabilities, and its advantageous structure provides it an extra edge in performing these dual functions. It is still a matter of investigation how exactly these decisions are being made by CHIP, inside the cells. A major question yet to be answered is, how CHIP, in combination with other chaperones recognizes the abnormal and to be degraded substrates inside the cytoplasm. Further work in this direction will indeed aid in understanding the possible ways of identifying cellular proteins on the basis of their structural and physiological features. Several studies have been done to explore the methods of discriminating the abnormal proteins from the healthy ones, whether they are to be modified, modulated or degraded. But, a clear answer is still far from our current understanding. Major focus of research over CHIP has been given to its roles in signaling, apoptosis and cell proliferation. But, a line of evidence clearly indicates that this molecule has equally important contribution in maintaining the cellular homeostasis and providing protection from various stresses, which are major factors causing protein aggregation and related neurodegenerative diseases. Few possible developmental and aging-related roles of CHIP has also been observed; still a lot of research is needed to be done to understand the physiological importance of CHIP in neurodevelopmental and aging processes.

## Key Questions and Future Perspectives

Maintaining a healthy proteome is a prerequisite of a living cell. To achieve this, cells are evolved with chaperone machinery and quality control pathways that maintain proteins into proper conformation and protect cells from toxicity generated in various pathological conditions (Yerbury et al., 2016). CHIP represents a protein which links all these pathways and machinery together. It not only helps chaperones to fold proteins, but also directs misfolded proteinaceous species for their proteasomal or lysosomal degradation. Interestingly, CHIP also regulates chaperones by ubiquitination and to more surprise, it also has self-ubiquitination property, which makes it capable of regulating its own turnover (Murata et al., 2001). Despite the potential that CHIP and other E3 ubiquitin ligases have, as therapeutic agents, more data is required, and further work is needed to be done, to understand the extent of E3 ubiquitin ligases in controlling various cellular processes. Chaperone-independent role of CHIP is still needed to be fully established, which can unveil its various other properties. How to achieve specificity, in the case of CHIP, is another criterion that should be worked upon, if this multifaceted ligase has to be used for therapeutic benefits. Being a central molecule in various processes, merely overexpressing it will also affect other pathways and substrates that could prove this strategy detrimental in place of being beneficial. So, besides looking for pathways and substrates, properties and strategies, that could increase specificity for intended substrate will undoubtedly open a new area for treating diseases that are difficult to handle, with our current treatment strategies.

Although, enough reports have been made to establish protective roles of E3 ubiquitin ligases in various neurodegenerative diseases, still no notable success has been registered so far to treat these life-threatening neuronal disorders. In one of our previous review articles, we have selected few such E3 ligases which have shown cellular protection against these pathological conditions (Upadhyay et al., 2015). CHIP, with an array of protein quality control functions, including regulation of chaperones and autophagic pathways, posits it as one prominent candidate, which has a huge potential to be targeted in the future therapeutic models of neurodegenerative diseases. The ability of CHIP to interact with numerous proteins and participation in multiple cellular pathways makes it one of the most interesting E3 ubiquitin ligase. CHIP, with described functions, bestows to be a possible future therapeutic target which could be exploited by the drug industry in the future. With such a significant contribution in cellular homeostasis, CHIP needs further attention of the scientific community to think and devise strategies to regulate multiple pathways of cellular importance by using this molecule. Focus on specific details and elaborated knowledge of underlying mechanisms will allow us to use CHIP as a therapeutic molecule in future.

## Author Contributions

VJ, AU, AA and RM, executed complete drawing of figures, AK provided critical inputs. AM formulated the entire concept of manuscript and designed the initial draft of figures. All authors reviewed the manuscript.

## Conflict of Interest Statement

The authors declare that the research was conducted in the absence of any commercial or financial relationships that could be construed as a potential conflict of interest.

## Abbreviations

AhR: aryl hydrocarbon receptor
AIF: apoptosis-inducing factor
Akt: AKR/J thyoma protein kinase
ALS: amyotrophic lateral sclerosis
APC: anaphase promoting complex
APP: amyloid precursor protein
AR: androgen receptor
ASK1: apoptosis signal-regulating kinase 1
AXL: receptor tyrosine kinase AXL
BAG: B-cell lymphoma-2 (BCL-2)-associated athanogene
BCR- ABL: break point cluster abelson murine leukemia viral oncogene
BMP: bone morphogenetic protein
CAD: caspase recruitment domain
CAP: chaperone-assisted proteasomal degradation
CARMA1: CAD containing membrane-associated guanylate kinase 1
CASA: chaperone-assisted selective autophagy
CDK5: cyclin-dependent kinase 5
CFTR: cystic-fibrosis transmembrane-conductance regulator
CHIP: C-terminus of Hsc70-interacting protein
CMA: chaperone-mediated autophagy
c-myc: cellular myelocytomatosis viral oncogene homolog
CtBP2: C-terminal binding protein 2
CYP3A4: cytochrome P450 isoform 3A4
CytP450: cytochrome P450
DLK: dual leucine zipper-bearing kinase
DNA Lig III: DNA ligase III
DNA pol B: DNA polymerase B
DUB: deubiquitylating enzyme
EGFR: epidermal growth factor receptor
eIF4E: eukaryotic translation initiation factor 4E
eIF5a: eukaryotic translation initiation factor 5a
ER: estrogen receptor
ErbB2: erythroblastic leukemia viral oncogene homolog 2
ERK: extracellular signal-regulated kinase
FGFR3: fibroblast growth factor receptor 3
FoxO1: fork head transcription factor 1
FoxP3: forkhead box P3
GCH1: guanosine-5′-triphosphate cyclohydrolase 1
GHR: growth hormone receptor
GR: glucocorticoid receptor
GUCY: guanylate cyclase
HECT: homologous to the E6-AP carboxyl terminus
HIF-1α: hypoxia-inducible factor-1 alpha
Hip: Hsc70 interacting protein
Hop: Hsp70/Hsp90 organizing protein
HSF1: heat shock factor 1
HspB8: heat shock protein B8
hTERT: human telomerase reverse transcriptase
ICER: inducible cyclic activated protein kinase (AMP) early repressor
IRE1: inositol requiring enzyme 1
IRF1: interferon regulatory factor 1
JNK: c-Jun N-terminal kinase
LC3: microtubule-associated protein 1A/1B-light chain 3
LKB1: liver kinase B 1
LRRK2: leucine-rich repeat kinase 2
MAPK: mitogen-activated protein kinase
MDM2: mouse double minute 2 homolog
MEKK2: MAPK/ERK kinase kinase 2
MIF: migration inhibitory factor
MKKS: McKusick-Kaufman syndrome protein
MLK3: mixed-lineage kinase 3
nAChRs: neuronal nicotinic acetylcholine receptors
NFkB: nuclear factor κB
NPM-ALK: nucleophosmin-anaplastic lymphoma kinase
Pael-R: Parkin-Associated Endothelin Receptor-Like Receptor
PI3K: phosphoinositide 3-kinase
PKCζ: protein kinase C ζ
PRMT5: protein arginine methyltransferase 5
PTEN: phosphatase and tensin homolog deleted on chromosome TEN
PTK6: protein tyrosine kinase 6
Raf1: rapidly accelerated fibrosarcoma 1
RING: Really Interesting New Gene
Ron: recepteurd’originenantais kinase
Runx1: runt-related transcription factor 1
SENP3: SUMO/sentrin protease 3
SEREX: serologic identification by recombinant expression cloning
SGK1: serum and glucocorticoid-regulated kinase-1
SirT6: Sirtuin6
Smads: Sma-mother against decapentaplegic
SOD1: superoxide dismutase1
Src: sarcoma viral oncogene homolog
SRC3: steroid receptor coactivator-3
STUB1: STIP1 homology and U-Box containing protein 1
Tal1/Sc1: T-Cell acute lymphocytic leukemia 1
TDP-43: TAR DNA-binding protein 43
TPR: tetratricopeptide repeat
TRAF2: TNF receptor-associated factor 2
TRAT: T cell receptor associated transmembrane adaptor 1
UFD2: ubiquitin fusion degradation 2
UPS: Ubiquitin Proteasome System
XRCC1: X-ray repair cross-complementing group-1.
